# Towards personalized dementia care through meaningful activities supported by technology: A multisite qualitative study with care professionals

**DOI:** 10.1186/s12877-021-02408-2

**Published:** 2021-08-21

**Authors:** Gemma Goodall, Kristin Taraldsen, Randi Granbo, J Artur Serrano

**Affiliations:** 1 Department of Mental Health, Norwegian University of Science and Technology, Trondheim, Norway; 2 Department of Neuromedicine and Movement Science, Norwegian University of Science and Technology, Trondheim, Norway

**Keywords:** dementia, immersive technology, person-centred care, nursing homes, reflexive thematic analysis, qualitative research methods

## Abstract

**Background:**

Person-centred care is widely recognised as important for helping people with dementia maintain a sense of self and purpose in life – especially for those living in care facilities. Despite this, most care practices still adopt a medical approach in which physical needs are prioritized over psychosocial well-being. Addressing the need to find ways of promoting person-centred approaches in care, this study explored care professionals’ reflections on a novel, technological intervention (SENSE-GARDEN) that combines multisensory stimuli and digital media to create personalized environments for people with dementia. The aim of this study was to explore the experiences of care professionals who had used SENSE-GARDEN for approximately 1 year.

**Methods:**

Three care homes in Norway, Belgium, and Portugal and 1 hospital in Romania used the SENSE-GARDEN with residents/patients with moderate to severe dementia over the course of 1 year. Qualitative data - including observations and interviews with 2 care professionals - were collected at the beginning of the study period from the Norwegian care home to explore initial impressions of the new SENSE-GARDEN room. At the end of the study period, 8 care professionals across the 4 facilities were interviewed for an in-depth exploration of their experiences. The two sets of data were analysed separately through reflexive thematic analysis.

**Results:**

At the beginning of the study period, the staffs’ focus was mainly on the novelty of the new SENSE-GARDEN room and how it provided opportunities for meaningful experiences. Post-intervention, the care professionals provided reflective accounts on how care could be delivered in alternative ways to standard practice. The themes generated from the post-intervention interviews were: “shifting focus onto personalized care”, “building and fostering relationships”, and “continuous discoveries”. Through delivering person-centred care, the professionals reported a sense of purpose and achievement in their work.

**Conclusions:**

Professionals from care facilities across 4 different countries highlighted the value of interventions such as SENSE-GARDEN as a way of creating opportunities to better know people with dementia. Thus, they experienced improved relationships and greater job satisfaction. However, delivering person-centred interventions is time-consuming, and future research should evaluate the feasibility of sustaining them on a long-term basis.

**Supplementary Information:**

The online version contains supplementary material available at 10.1186/s12877-021-02408-2.

## Background

The prevalence of dementia is increasing on a global level, with the number of people living with dementia approximately doubling every 5 years [1]. Whilst there are varying types of dementia, the progressive nature of the disease means that many people often experience changes in behaviour, memory, language, and/or communicative abilities. For individuals living in and transferring to care homes, the added uncertainty around being cared for in an unfamiliar setting can cause further threat to preserving a sense of self, maintaining social relationships, and living a meaningful life [2, 3]. As such, it is important to identify ways of optimizing care environments so that the well-being of residents with dementia is supported.

Recent evidence suggests that the quality of care provided by staff has a lasting impact on the quality of life for care home residents [4, 5]. Over the last two decades there has been an emphasis on providing person-centred care towards people living with dementia, in which needs such as comfort, attachment, inclusion, occupation and identity are given the same priority as medical or physical needs [6, 7]. Furthermore, there is growing acknowledgement that meaningful, individually targeted activities should be integrated into the care of residents living in care institutions [8, 9]. However, despite a general consensus for a person-centred approach to dementia care across Europe, the practice of delivering person-centred care can vary greatly due to the lack of support that professionals may receive from their organizations [10]. If staff are expected to deliver person-centred dementia care, it is vital that they are provided the tools and opportunities to do this. In their global action plan on the public health response to dementia 2017–2025, the World Health Organization underlined that staff should be supported in delivering evidence-based treatment and care [11]. Such calls to action resonate in national legislation. In Norway, for instance, the Dementia 2020 plan recognised the importance of a person-centred approach in care services and placed an emphasis on providing meaningful activities in the care of people living with dementia [12].

Despite these calls to action, a recent scoping review of the needs and experiences of people living with dementia in nursing homes found that one of the most commonly occurring experiences of residents was boredom [13]. Furthermore, a recent study of people living with dementia in Norwegian care homes found that residents desired more opportunities for meaningful relations and activities [14]. Both in Norway and internationally, the delivery of dementia care still tends to be based on a medical model of care which is oriented towards physical needs rather than psychosocial needs [15–17].

Previous studies have found that delivering one-to-one, meaningful activities on a regular basis in care homes can be challenging due to factors such as lack of opportunities, lack of staff resources, and the workload of care staff [18, 19]. Additionally, Harmer and Orrell [18] reported that staff lacked sufficient skills in identifying and delivering activities that residents with dementia could engage in. More recent evidence has identified numerous barriers of implementing person-centred care in practice, which include issues such as time constraints, staff turnover, negative attitudes, poor relationships, lack of consistency in care personnel, a lack of understanding of dementia, and a lack of autonomy and empowerment towards residents [20–23]. With the large prevalence of people living with dementia and importance of supporting personhood – especially in later stages of the disease – it is necessary to investigate ways in which care staff can be supported in providing person-centred care through meaningful interactions.

Technological solutions may be a means of helping caregivers (both familial and professional) facilitate meaningful activities for people with dementia. Multimedia technologies are increasingly being used to create digital life storybooks and apps in which personal photographs, videos and music are combined to facilitate joint reminiscence [24–26]. Using these kinds of multimedia tools to explore the narratives of people with dementia can promote well-being and social engagement amongst people with dementia, families, and care staff [27]. Furthermore, a recent literature review on the use of technology in creating individualized, meaningful activities for people living with dementia suggests that digital technologies can be promising in terms of improving well-being and promoting relationships with others [28]. However, findings from the review also suggest that further work is needed on how to implement technologies into care environments.

### SENSE-GARDEN

This study is part of an interdisciplinary European project, SENSE-GARDEN [29]. The SENSE-GARDEN is a room that combines various technologies, such as projected films and imagery, music, and scents to provide numerous activities all based on the life story of the individual. It is designed to be a tool by which caregivers can easily access and engage with the life stories of people with dementia and is thus used as a means of delivering a person-centred intervention. SENSE-GARDEN has evolved through several steps where target users (including people with mild dementia), clinicians, and researchers have collaborated [30]. In a recent study of SENSE-GARDEN use amongst people with dementia and their friends and family members, findings suggested that the intervention can stimulate emotional experiences, preserve narrative identity, and foster interpersonal relationships [31]. However, given the novelty of this technological intervention, the input of care professionals regarding the practicality of SENSE-GARDEN is essential. Understanding how professionals feel about using SENSE-GARDEN in day-to-day care will provide insights into the potential of integrating its use into care environments outside the context of a research project.

### Aim

The overall aim of this study was to explore the perspectives and experiences of care professionals using the SENSE-GARDEN in practice. The specific research questions falling under this aim were (a) How do care professionals use the SENSE-GARDEN with care home residents and hospital patients with moderate to severe dementia? And (b) What do care professionals consider as benefits and challenges of using the SENSE-GARDEN in practice? Through addressing these questions, it is hoped that an understanding of how the SENSE-GARDEN may be integrated into care environments in the future can be gained.

## Method

### Study design

In this study, 3 care homes in Norway, Belgium, and Portugal and 1 hospital in Romania used the SENSE-GARDEN with residents/patients with moderate to severe dementia over the course of 1 year. To gain an in-depth understanding of the care professionals’ experiences, a qualitative approach was used.

The study was split into two parts. The rationale for doing this was to first explore how two members of staff at the Norwegian care home were using the new SENSE-GARDEN room and intervention with residents during the first week of the intervention period, and to explore their initial reactions to the novel addition to the care facility. These two members of staff were interviewed again in the second part of the study after a year, along with 6 other care professionals across the test sites in Belgium, Portugal, and Romania. Interviewing them again along with care professionals at the other test sites allowed us to capture reflections of using the SENSE-GARDEN over a longer period of time (approximately 1 year).

The first part of the study captured initial impressions towards the newly built SENSE-GARDEN room at the care home in Norway. To observe how the staff were using the new SENSE-GARDEN room and delivering the intervention to residents, the first author observed SENSE-GARDEN sessions at the Norwegian test site before conducting a semi-structured, face-to-face interview with two members of care staff in September 2019. The field notes and transcripts were analysed thematically and informed the design of the second part of the study.

The second part of the study took place in January 2021 and explored care professionals’ reflections and experiences having used SENSE-GARDEN for just over a year. At the time of these interviews, the SENSE-GARDEN at each site had been in operation for just over a year. Eight care professionals across all four test sites (Norway, Portugal, Belgium, and Romania) were interviewed using Zoom Video Communications Inc. (Zoom) and Microsoft Teams.

### Setting

**Fig. 1:**
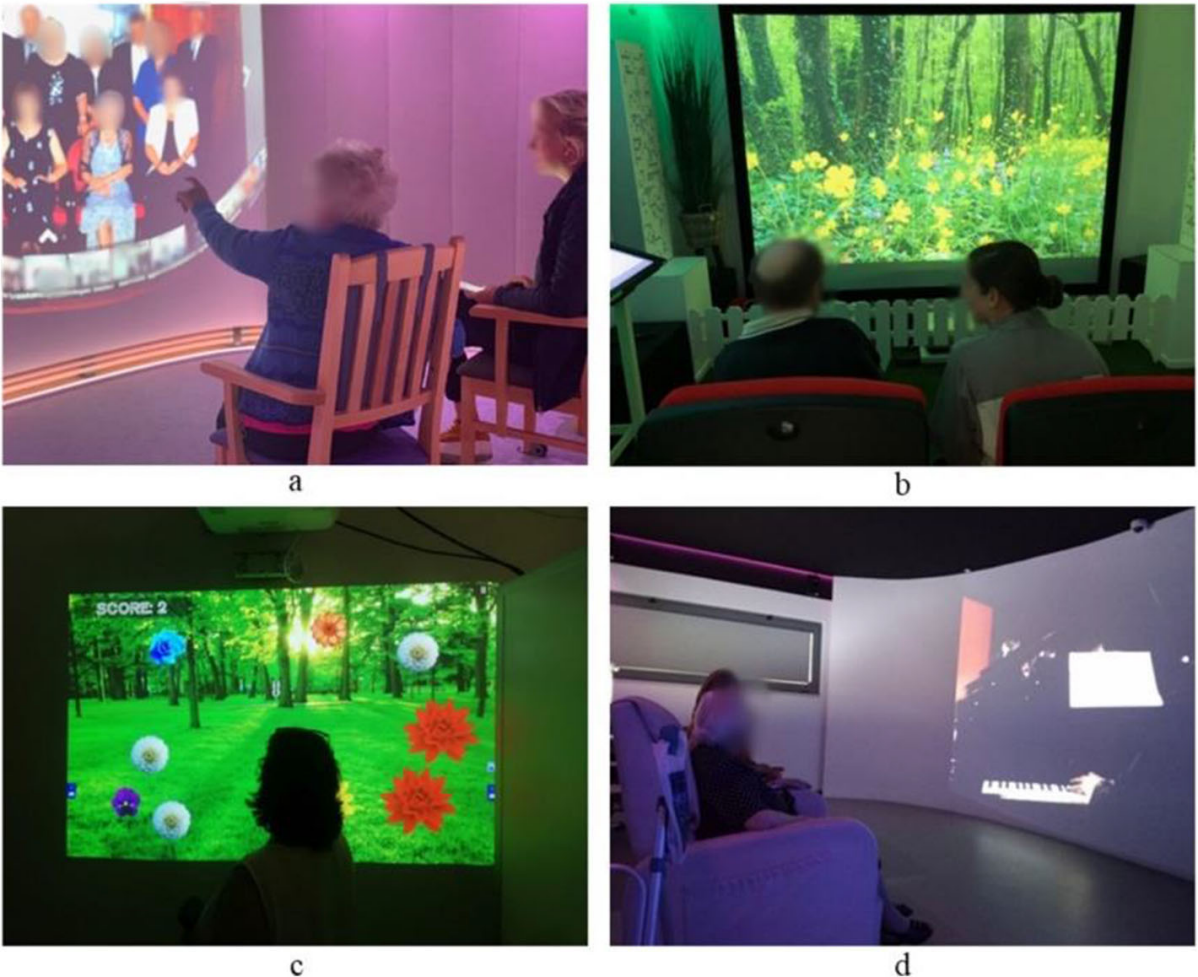
The SENSE-GARDEN room in Norway (**A**), Belgium (**B**), Romania (**C**), and Portugal (**D**)

The study involved professionals at care homes in Norway, Portugal and Belgium, and a hospital in Romania. A photograph of the SENSE-GARDEN room at each of the four test sites is shown in Fig. 1.

In Norway, the SENSE-GARDEN room is located in a municipality-based care home in a remote town with under 10,000 inhabitants. In Portugal, the room is in a care home belonging to a large non-profit organization in a city of over 500,000 inhabitants. In Belgium, the room is located in a care home belonging to a large care organization in a small village. In Romania, the SENSE-GARDEN is located in a hospital’s rehabilitation centre in a large city of over 1.8 million people.

### Participants

All participants in this study were professionals working at each of the test sites where the SENSE-GARDEN room was located. In the first part of the study, the first author contacted the manager of the Norwegian care home to arrange a visit for observing SENSE-GARDEN sessions and conducting an interview with 2 care professionals. The purpose of these interviews was to capture initial responses and thoughts towards the newly built SENSE-GARDEN room. In the second part of the study, the first author contacted care professionals at each of the SENSE-GARDEN test sites to invite them for an interview on their experiences of using the intervention with residents/patients. The first author also asked the professionals for contact details of any other members of staff who had used the SENSE-GARDEN. Participants were only included in the study if they had used the SENSE-GARDEN with residents/patients with dementia. Given the small number of care professionals using the SENSE-GARDEN at each care facility, a total of 8 participants across 4 test sites were interviewed. These 8 participants included the 2 care professionals that were interviewed in part 1 of the study, meaning that the 2 care professionals from Norway were interviewed twice (once in 2019 and again in 2021). An overview of the participants’ demographic information is given in Table 1.

**Table. 1:** Participant demographics

Test Site	Gender	Age	Job Title	Years of experience in dementia care	Educational background
1	Female	37	Psychologist	4	Psychology
1	Female	33	Sociologist	5	Sociology and Social Work
1	Female	58	Board member of care organization	10	Social politics and creative leadership
2	Female	37	Occupational therapist	16	Occupational therapy
3	Female	51	Researcher in Physical and Rehabilitation Medicine	25	Rehabilitation Medicine
3	Female	41	Clinical psychologist	10	Rehabilitation
4	Female	37	Nurse	14	Nursing
4	Female	40	Institution manager	16	Nursing

### SENSE-GARDEN as an intervention

The SENSE-GARDEN is a room with technological solutions used to deliver individualized interventions to people with moderate to severe dementia. The dedicated room is primarily designed for interaction between the person with dementia and a professional member of staff at the care facility (referred to as a “formal caregiver”). Family members (referred to as “informal caregivers”) help the formal caregiver prepare the sessions by providing photographs, films, and information about the person with dementia. Additionally, they are encouraged to join the SENSE-GARDEN sessions if they are available to do so. However, the current study focuses only on the perspectives of the formal caregivers.

The approach to using the SENSE-GARDEN space for facilitating an individualized intervention for people with dementia borrows techniques from reminiscence therapy, encouraging the individual to recall and reflect on people, places, and events from their lives [32]. Reminiscence therapy has been widely used in dementia care since the late 1970 s [33]. In recent years, the use of technology to complement and aid the facilitation of reminiscence activities has been explored with promising results, especially with regards to promoting social interaction and providing the opportunity to access a vast amount of media from both the past and more recent events [24–26, 34].

### The process of personalizing visits

**Fig. 2:**
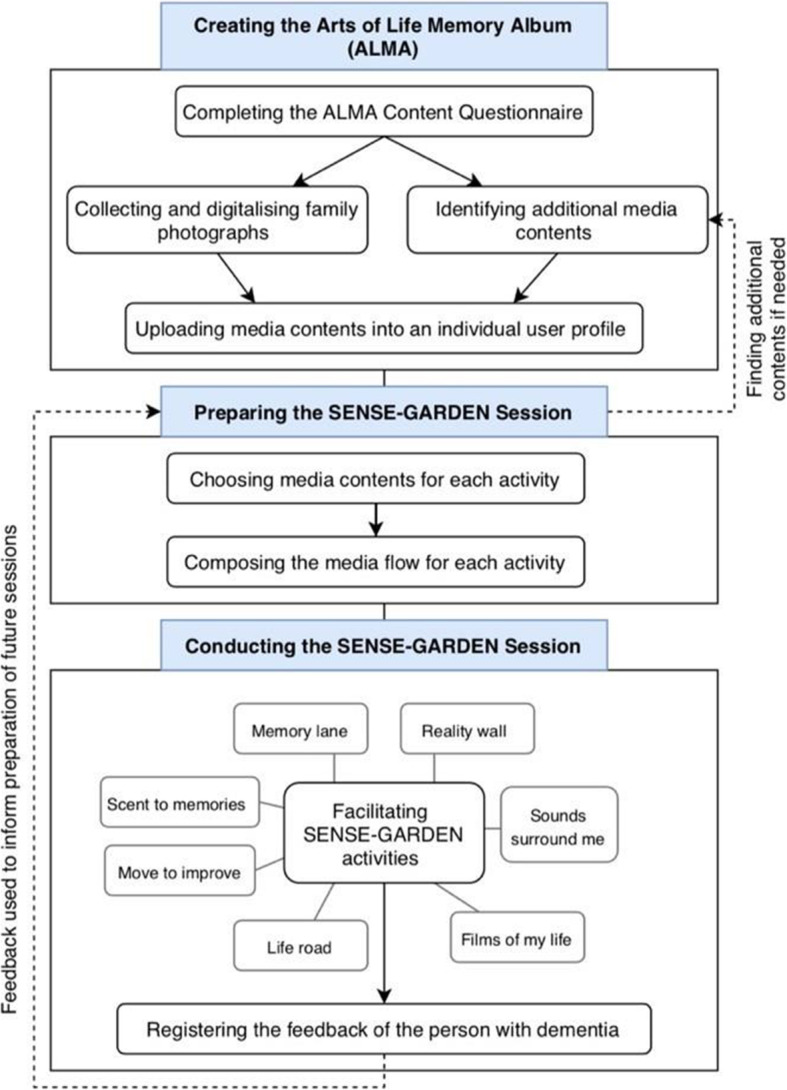
Overview of preparing and facilitating SENSE-GARDEN sessions

A prerequisite to using the SENSE-GARDEN is creating an individual user profile for each person with dementia, referred to as an “Arts of Life Memory Album” (ALMA), so that the sessions can be tailored to each user. This involves the family of the resident providing information by filling out a questionnaire about the individual. The questionnaire, designed by researchers in the SENSE-GARDEN project, consists of 75 closed and open-ended questions relating to topics such as family history, working life, leisure, and emotional needs. In addition to completing the questionnaire, family members are also asked to provide any photographs and videos that are significant to the person with dementia. An overview of this process is shown in Fig. 2.

**Fig. 3:**
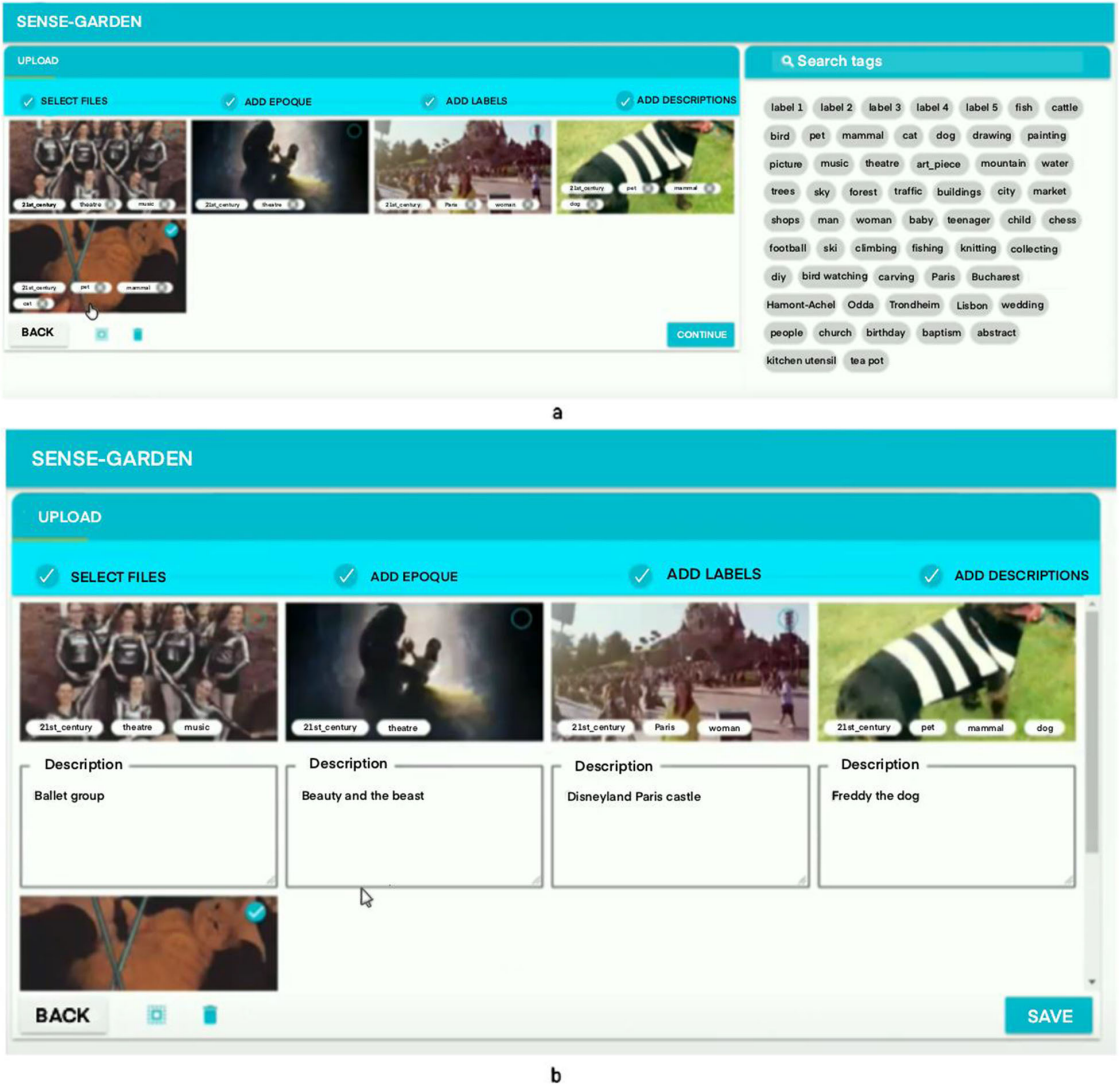
Uploading media contents and assigning labels (**A**) and assigning descriptions (**B**)

Media contents consisting of pictures, videos, and music are uploaded to a user profile using an online platform developed by the SENSE-GARDEN team (see Fig. 3). The uploaded media is assigned “labels” that describe its content e.g. “dog” or “forest”. The purpose of adding labels is to make it easier for caregivers to find media contents related to a specific theme or part of the individual’s life history when planning future sessions. Free-text descriptions of the media can also be added to provide caregivers with more detailed contextual information about the media.

**Fig. 4:**
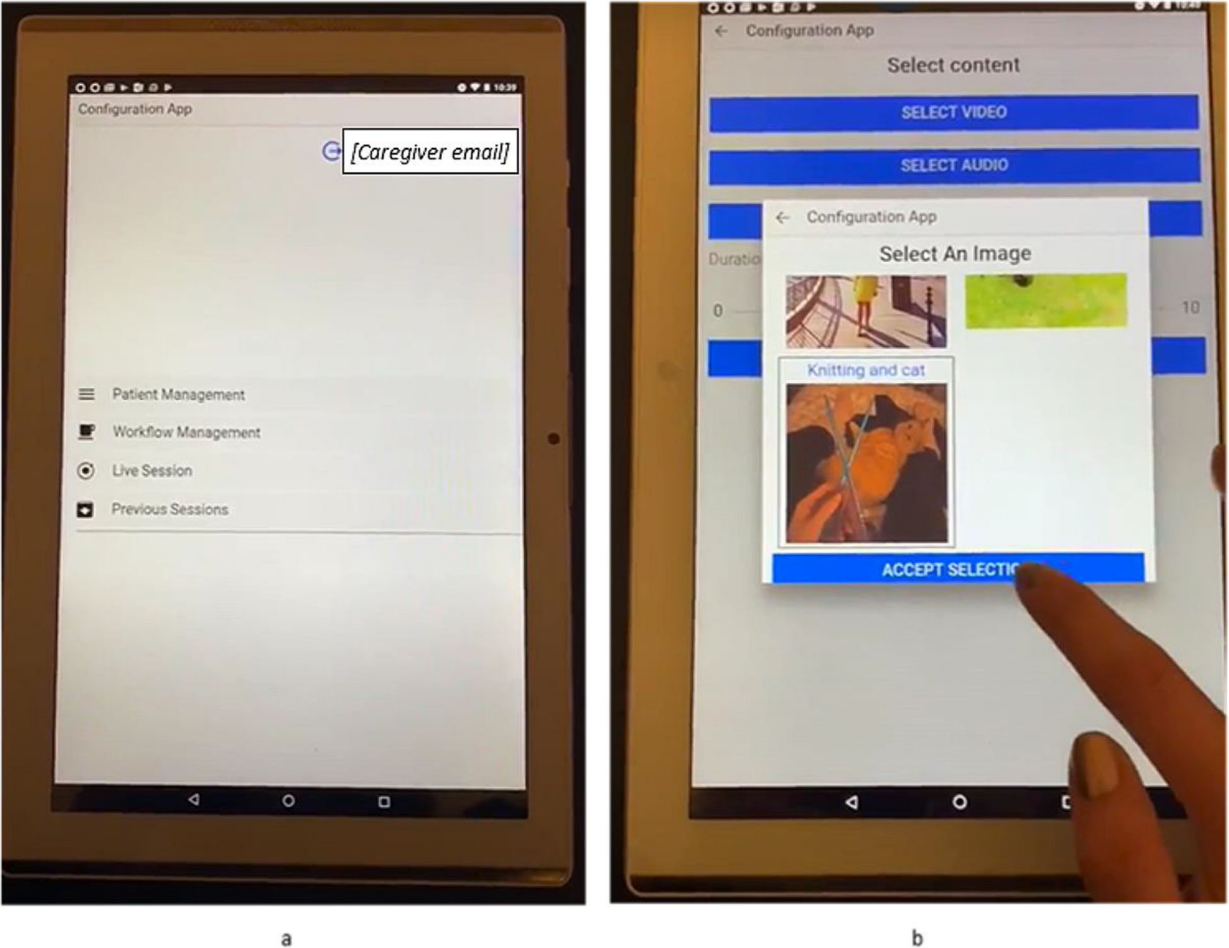
Using the SENSE-GARDEN Tablet app. The homepage of the app (**A**) and creating a media flow (**B**)

Once the ALMA has been created, its contents are then used by the formal caregiver to compose media flows, which are sequences of photos, videos and music that are relevant to the individual. A tablet app, designed by the SENSE-GARDEN technical team, is used by the formal caregiver to create these flows of media contents for each of the activities within the SENSE-GARDEN space (see Fig. 4). The flows are then used in the SENSE-GARDEN sessions.

### Facilitation of the sessions

**Fig. 5:**
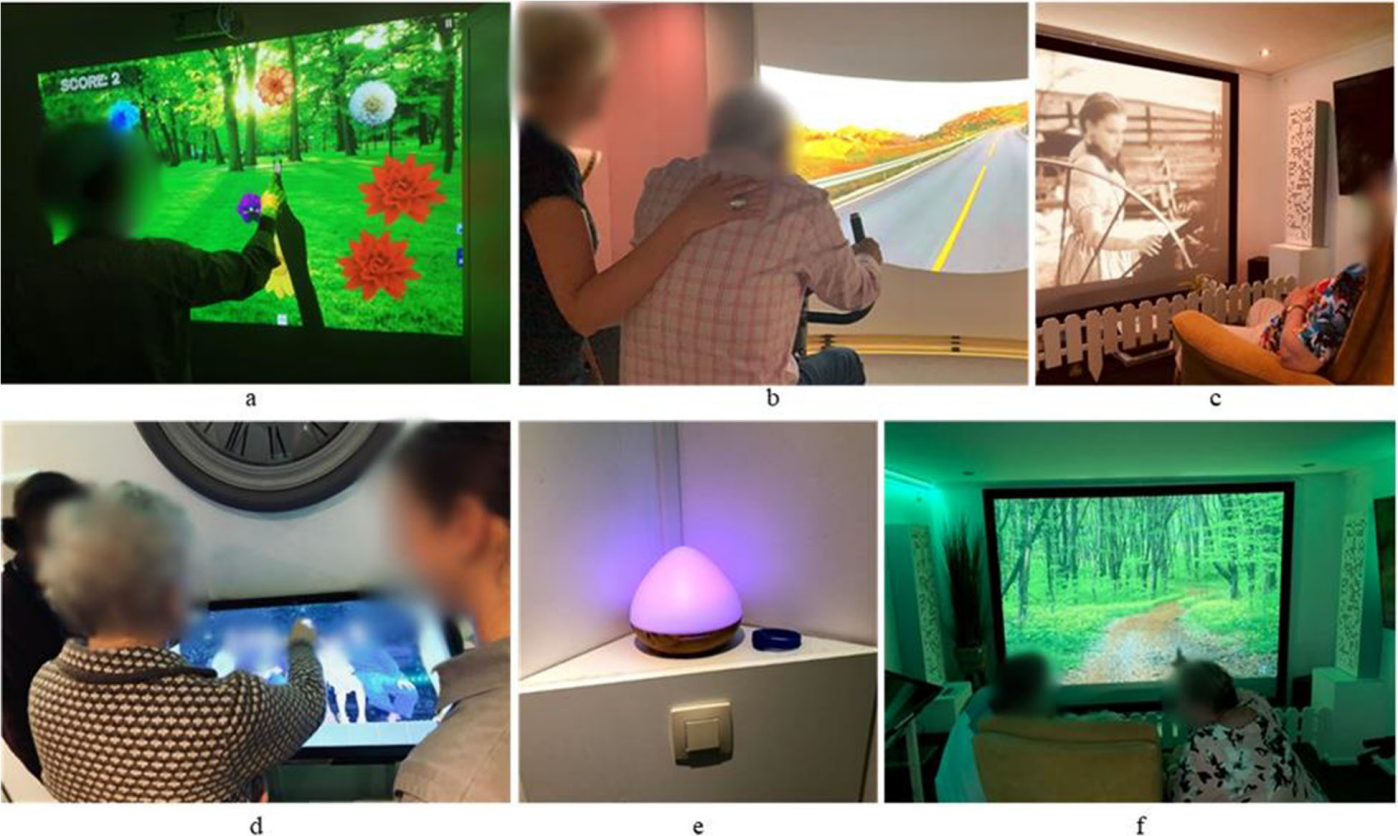
Activities within the SENSE-GARDEN: Move to Improve (**A**); Life Road (**B**); Films of My Life (**C**); Memory Lane (**D**); Scent to Memories (**E**); Reality Wall (**F**) Reprinted from Goodall G, Andre L, Taraldsen K, Serrano JA. Supporting identity and relationships amongst people with dementia through the use of technology: A qualitative interview study. International Journal of Qualitative Studies on Health and Well-being. 2021; 16(1):1,920,349

For a session, one person with dementia enters the SENSE-GARDEN room with a caregiver. Family members are encouraged to join the sessions if they are available to do so. However, most of the sessions consist of just two individuals – the person with dementia and the professional member of care staff. Together, the person with dementia and caregiver interact with the various activities and stimuli in the space (see Fig. 5). These include family photographs, project films and images, surround sound music, olfactory stimuli delivered used a scent dispensing system, an exergame, and a stationary bike activity accompanied by a film of a familiar place e.g. the individual’s hometown. Each session lasts for approximately 30–60 min.

**Fig. 6:**
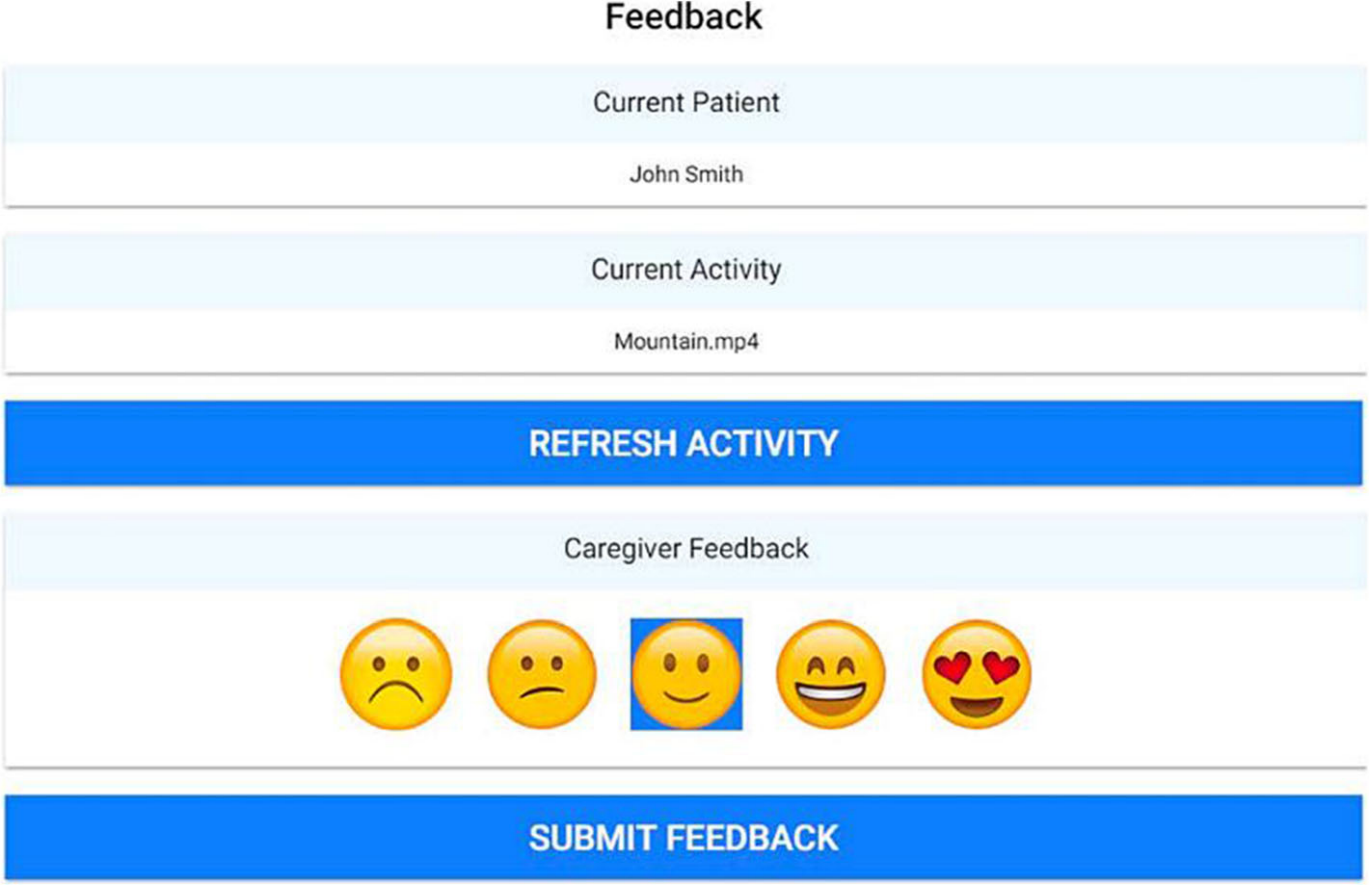
Giving feedback via the app

The SENSE-GARDEN app is used by the formal caregiver to register feedback in response to the media contents used in the session. This feedback is based on both verbal and non-verbal reactions of the person with dementia and is used to improve the selection of media contents for subsequent SENSE-GARDEN sessions. The caregiver registers this feedback by choosing from emoticons in the app indicating the overall response of the person with dementia during each activity (e.g. Memory lane). This is shown in Fig. 6. The caregiver may also write unguided feedback in text boxes shown in the app.

### Data collection

Data was collected for this study in two parts. In both parts, the data was collected by the first author, who was a PhD candidate within the SENSE-GARDEN project at the time of data collection.

#### Part 1: Beginning of SENSE-GARDEN intervention

To gain insight into how the care staff facilitated the SENSE-GARDEN sessions, the first author (GG) visited the Norwegian test site for three consecutive days. During this time, she observed 4 SENSE-GARDEN sessions facilitated by a member of care staff at the facility. Additionally, in order to reflect upon the amount of effort required from staff to identify and prepare media contents for the session, GG observed the staff member conducting these preparations. The approach to observing the SENSE-GARDEN sessions was not to solely focus on interactions between staff and residents, but to also take note of actions taken regarding the use of technology. The first author, familiar with the technology within SENSE-GARDEN, was able to focus on how the care staff were using the SENSE-GARDEN system and app. Reflective field notes were made at the end of each day, as not to hinder the atmosphere during sessions or time spent with the care staff [35].

On the final day of the visit, an in-depth, semi-structured interview was conducted with two members of the care staff at the facility who had been observed using SENSE-GARDEN. The observations and field notes collected during the week were used to inform the design of the interview guide. Questions were open-ended and asked the care staff about their first impressions of the newly built SENSE-GARDEN room, their experiences of using the SENSE-GARDEN together with residents, the process of creating individualized user profiles, and the anticipated feasibility of integrating the SENSE-GARDEN into routine care (see Supplementary Material A for the interview guide). The observations and field notes also provided useful, contextual background information that helped the interview flow naturally in a conversational style.

#### Part 2: Interviews after 1 year of SENSE-GARDEN use

To capture the experiences of professionals involved in the project, 6 care professionals across the test sites in Portugal, Belgium and Romania were interviewed at the end of the intervention period in January 2021. Additionally, the 2 care professionals at the Norwegian test site who participated in the first part of the study were interviewed for a second time, so their reflections from having used SENSE-GARDEN for 1 year could also be included. Thus, a total of 8 participants were interviewed in 2021. The semi-structured interview guide (see Supplementary material B) included open-ended questions that were similar to part 1 of the study. However, the focus of this interview was more on capturing the professionals’ reflections of using SENSE-GARDEN for over 1 year, and to assess whether they deemed it suitable for care on a long-term basis. Due to this being a multisite study, interviews were conducted over Zoom and Microsoft Teams. The interviews were conducted by the first author (GG), who had visited all 4 SENSE-GARDEN spaces during the project period. All interviews were conducted in English. Prior to the interviews in both stages of data collection, the interviewer assured the participants that they could be completely honest in their opinions about SENSE-GARDEN.

All interviews in parts 1 and 2 were audio recorded. Informed consent was collected prior to the interviews. All participants agreed to having the interviews recorded. In order to allow the care professionals to speak freely, the test sites and participants were pseudonymized by GG. The interviews were transcribed verbatim by GG. There was a total of 4.5 h of recording with 47 pages of transcripts.

### Analysis

The transcripts were analysed using reflexive thematic analysis, which aims to generate themes that reflect a pattern of shared meaning around a central organizing concept [36–38]. Reflexive thematic analysis (RTA) acknowledges the active role of the researcher in the production of knowledge and embraces researcher subjectivity as a resource [38]. In the present study, RTA was primarily conducted by GG, who was able to reflect upon her experiences at each of the 4 test sites and her time spent inside the SENSE-GARDEN with both care staff and residents with dementia at the Norwegian test site in particular.

Transcripts from parts 1 and 2 of the study were initially analysed separately. Analysis of the transcripts took place shortly after the interviews were conducted, meaning that the transcript in part 1 of the study was analysed in winter 2019, and the transcripts from part 2 of the study were analysed in January 2021. The same group of authors analysed the transcripts in both parts of the study and the same analytical procedure was taken.

First, familiarization of the data took place through repeated listening of the audio recordings of the interview and repeated reading of the transcripts. During this process, initial notes and ideas about the data were made. All authors shared their initial ideas with one another. Data was then coded in an inductive and semantic manner by GG. Meaning was made from the transcript data in a “bottom-up” approach, rather than approaching the data with preconceived concepts or theories [38]. The generated codes were then used to construct initial themes. These themes were developed with the research questions in mind, as well as being conscious of how these themes could provide implications for meaningful dementia care practice beyond the context of SENSE-GARDEN. GG suggested ideas for initial themes, which were reviewed by all co-authors. Discussion took place regarding the meaning of each theme and how the themes related to the research questions at hand. The defining and naming of themes were also decided through joint discussion between all authors, during which the scope and focus of each theme was determined. During the discussion of data collected during part 2 of the study, the authors also reflected on how the new themes compared with those generated in the transcripts from part 1 of the study. Finally, the writing up of themes was conducted primarily by GG, who, during the writing process, reflected on the aim of the study, research questions, field notes and early familiarization of the transcripts to ensure the final themes and their portrayal remained close to the data and the research questions at hand [38]. All co-authors took an active and critical role in ensuring that the manuscript portrayed a clear narrative about the data.

## Results

The interview conducted in 2019 (part 1 of the study) captured first impressions towards SENSE-GARDEN, and the interviews conducted in 2021 (part 2 of the study) captured more detailed accounts of the professionals’ reflections after having used the SENSE-GARDEN for over a year. The overall impression between the two sets of data is that the professionals’ focus had shifted away from novelty of the new SENSE-GARDEN space and instead towards what can be achieved using SENSE-GARDEN. During the interviews in part 2 of the study, the professionals across all test sites reflected on the process of using SENSE-GARDEN as a way of being able to deliver personalized care and adding value to their work.

The main results and themes from Part 1 of the study are discussed briefly below, followed by a detailed description of the results, themes and subthemes generated in Part 2 of the study.

### Part 1: First impressions of SENSE-GARDEN

**Fig. 7:**
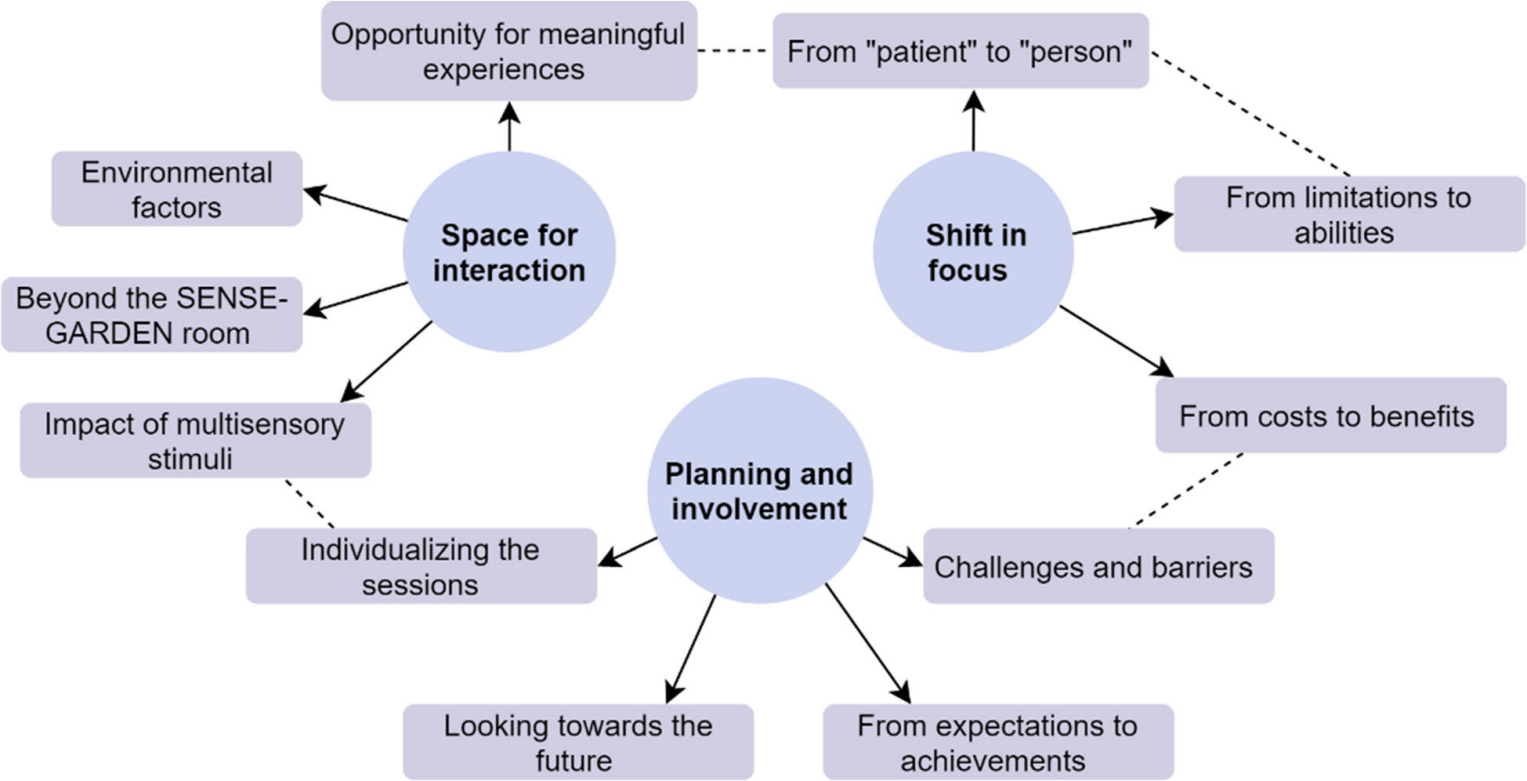
Thematic map of themes and subthemes generated from interview conducted in 2019

The findings from the interview with care staff at the beginning of the intervention period were positive overall, although the technological aspects of the SENSE-GARDEN were not quite finished. Three themes were generated from the interview conducted in 2019: space for interaction, shift in focus, and planning and involvement. These themes and subthemes are shown in Fig. 7, in which interactions between subthemes are shown using dotted lines.

The theme “Space for Interaction” captures the two professionals’ excitement towards the new SENSE-GARDEN room, and they said that residents were likewise impressed and intrigued by the new addition to the care home. The staff members responded positively to the physical features of the room and felt that it was a space in which opportunities for meaningful experiences could be created. Spending time inside the SENSE-GARDEN together with residents shifted the staff members’ views of each resident in terms of their identities and their capabilities, which is demonstrated in the theme “Shift in Focus”. By engaging with the life story of the resident using SENSE-GARDEN, the staff were able to shift their attention away from dementia and instead focus on the person as an individual:

You see the person and not the patient… and that’s a big difference

I think when you see the photos and you get their story… you see the person in a whole different way.

They also discussed technical problems that they had encountered, and how these caused frustrations. These issues are reflected in the theme “Planning and Involvement”, which highlights the importance of including care staff in the outset of testing a new interview for care. However, despite the issues they had encountered, both members of staff expressed enthusiasm about the potential benefits that SENSE-GARDEN may have on both staff and residents in the future:

I am quite excited that it actually is the way we planned it to be, but the space inside, it’s much more than you can imagine

I’m really excited about this and I think it will make a different for people with dementia and I hope that the project is successful and that we will manage to use this afterwards

### Part 2: Reflections after 1 year of SENSE-GARDEN use

**Fig. 8:**
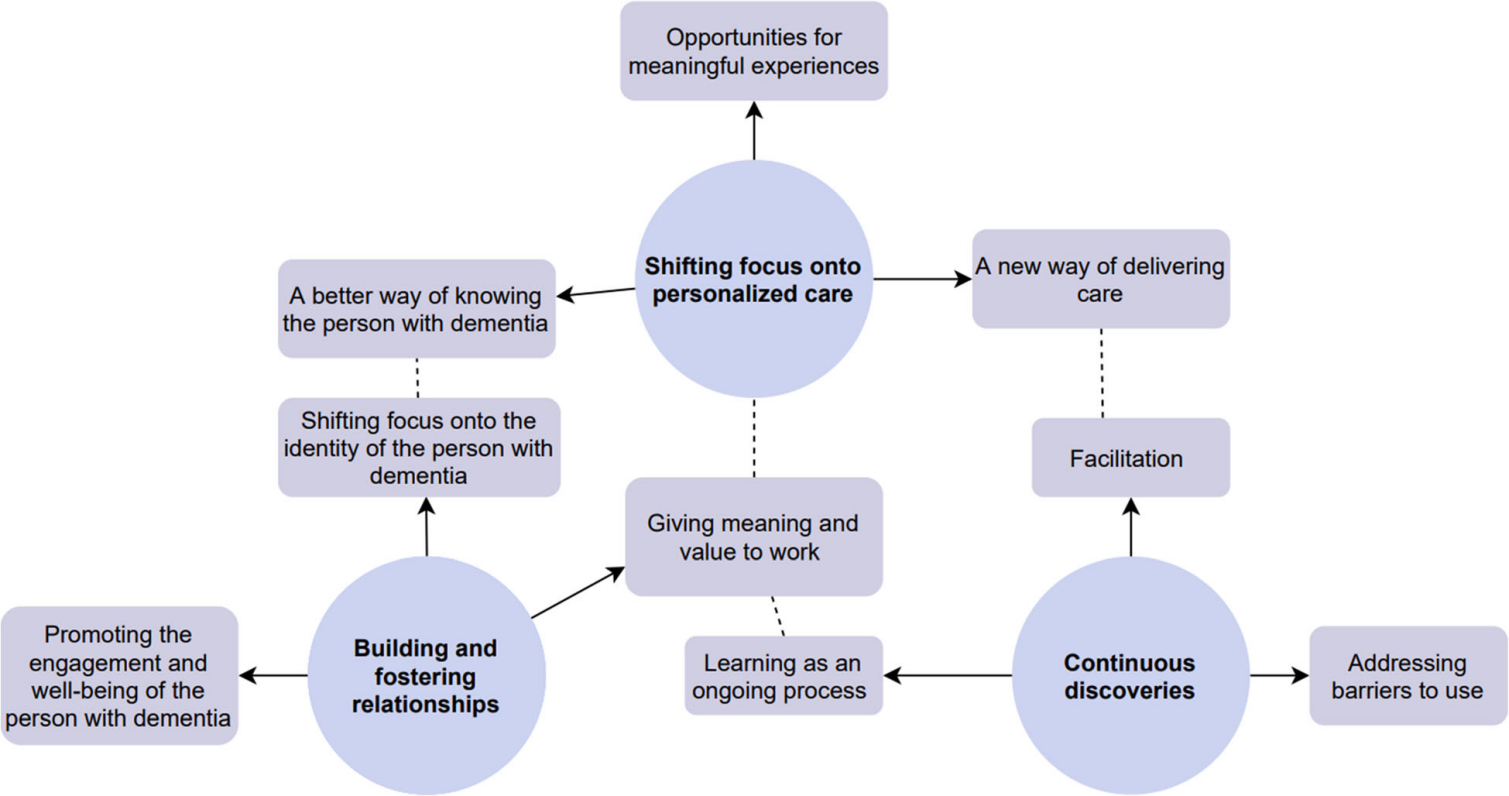
Thematic map of themes and subthemes generated from interviews conducted in 2021

Three themes were identified through analysis of the transcripts of the interviews conducted in 2021: shifting focus onto personalized care, building and fostering relationships, and continuous discoveries. A thematic map is shown in Fig. 8. Dotted lines are used to indicate the interaction between the different themes and subthemes. For example, the subthemes “A new way of delivering care” and “Facilitation” are under different themes but are related in the sense that the both subthemes concern the important role of the care professional.

### Theme 1: Shifting focus onto personalized care

One of the most prominent remarks about SENSE-GARDEN was the fact that the concept is based entirely on the life story and interests of the individual with dementia. The theme “shifting focus onto personalized care” captures the overall sense that the care professionals were able to approach care in a more playful and personalized manner compared to traditional care approaches. This theme has 3 subthemes: (1) “a new way of delivering care” relating to how professionals saw SENSE-GARDEN in relation to other approaches, (2) “a better way of knowing the person with dementia” reporting on how the professionals felt that SENSE-GARDEN is a tool for engaging with the life story of the individual, and (3) “opportunities for meaningful experiences” reporting on the professionals’ accounts of sharing meaningful, positive moments with residents/patients with dementia.

### A new way of delivering care

 During the interviews, the professionals reflected on the way in which the delivery of care in SENSE-GARDEN compared and related to other approaches such as cognitive stimulation, Snoezelen, and group reminiscence therapy. The care professionals felt that SENSE-GARDEN differed from these because more focus is placed on the specific interests of the individual:

It is focused on the participants (with dementia), their lives, and this is the most important intervention we have in [care home]

“It’s so personal, you know their life story and their history… I think this is more adapted to people with dementia than some other activities or therapies”.

Whilst professionals commented that they did already have activities tailored to the individual residents and patients, they felt that SENSE-GARDEN was different in the sense that it was more personal:

“Now I work with what the patient gives me from his personal experience, from his familial history… I work with the user’s materials in SENSE-GARDEN… and we put this material into an interactive form”.

The approach to the facilitation of the sessions was also compared to other therapies. Whereas more traditional approaches such as cognitive training have structured tasks, SENSE-GARDEN is more flexible in the sense that a session does not have to follow a set guideline and can be adapted based on the preferences of each person with dementia. This gives the caregiver more freedom to conduct a session in a way that they find more “comfortable”:

“Here we take a somehow playful approach, and in a very leisurely manner… so it’s more comfortable for everybody to work in this manner… it’s different and more life-like than formal training or cognitive training.”

In this way, SENSE-GARDEN can be seen as a way of providing caregivers more possibilities to conduct meaningful, leisure activities with the person with dementia. With this being said, SENSE-GARDEN was also seen as a good addition to other therapies. In one care home, an occupational therapist commented that SENSE-GARDEN is a good addition to the therapy that the residents get. In another test site, a psychologist remarked:

SENSE-GARDEN is not the solution to all problems with dementia, but it’s a good tool to work with others and with the professionals we have

One nurse at a different care home expressed her concerns that there is not enough emphasis placed on caring for the psychological well-being of residents with dementia:

I think the majority of therapy interventions or healthcare is oriented to physical aspects, and the psychological aspects are not taken very well care of… this causes a lot of problems.

This concern is in line with current literature on this topic and highlights the importance of providing care that can meet psychological needs as well as physical needs.

### A better way of knowing the person with dementia

The care professionals expressed their opinions on how SENSE-GARDEN could be used as a way of engaging with person with dementia and thus getting to know them in a better way compared to usual care:

I think it’s important for them to be involved in this kind of activity and to feel that someone is there who is attentive to their problems and attentive to their own existence

Additionally, a caregiver noted the importance of residents getting to know her so that they could more easily express themselves:

They don’t remember what they like so we have to adapt [the sessions] after a while and get to know them better… it’s easier to express themselves when they get to know me…. People with dementia don’t remember what they like, so I see it in their reaction, actually. I see how they react when I play different music and then I adapt it to each one of them.

This reciprocal process of expressing and learning provides caregivers an opportunity to get to know residents and patients in a way that might not be as easy in the usual care environment. This was seen to have benefits outside of the SENSE-GARDEN sessions:

The better you know the person with dementia, the better you can react to this person

Additionally, the process of collating information and preparing the sessions aided the connection between staff and residents/patients. The staff also commented on the fact that family members expressed they knew more about the residents through helping in the collection of biographical material and planning of sessions:

I also discovered for the family it’s a very nice thing to search for the information together. Many family members told me “Oh it was so nice to go through the history of my father or my mother” and see the pictures and learn about their own parents.

### Opportunities for meaningful experiences

During the interviews the professionals often gave accounts of moments they had experienced together with the residents/patients. SENSE-GARDEN provided opportunities to explore memories and stories of people’s lives together, and this was considered to be important:

I think that sharing these things is very important. They can work through their own memories alone at home, but it’s not the same. When you share these things, your memory, your life, and when you share any experience like this one, going to Paris together… it’s very important

The caregivers recalled strong reactions to moments inside the SENSE-GARDEN which were considered as meaningful for the residents/patients. For example:

One lady with dementia, she is fond of art, and I made a session with a great artist… we had the pictures and I put on very good music to it, and I could see that she closed her eyes and she was just enjoying that much that she was… she said that she had an inner journey

There was also a report of the information learned about a resident within SENSE-GARDEN being used to create an opportunity outside of the care facility. In a unique case, a professional caregiver took a resident to their old neighbourhood for coffee:

At the end of SENSE-GARDEN… [the care professional] went with this participant to a part of [city] where the participant had lived their whole life. She went with him… after all the sessions, after they talked about his life… he really liked it

Whilst opportunities to go on outings with individual residents are not easy to come by due to time constraints within care practice, the above quote serves as an example of how new knowledge on the resident had resulted in an opportunity for a meaningful experience beyond the context of SENSE-GARDEN.

### Theme 2: Building and fostering relationships

The theme “building and fostering relationships” reflects the process of building relationships over the course of using SENSE-GARDEN together with residents and patients. Spending time with the person with dementia inside the SENSE-GARDEN, interacting with the residents as individual persons instead of just “patients”, and investing time into making the ALMA all contributes towards the caregiving relationship. This theme has 3 subthemes: (1) “shifting focus onto the identity of the person with dementia” reporting on how caregivers were able to interact with residents and patients on a more personal level, (2) “promoting the engagement and well-being of the person with dementia” reporting on how the professionals noticed increased engagement and improved well-being as a result of SENSE-GARDEN use, and (3) “giving meaning and value to work” reflecting the sense of achievement and purpose that the professionals felt through using SENSE-GARDEN.

### Shifting focus onto the identity of the person with dementia

As found during the interviews in 2019, the professionals in the 2021 interviews spoke about how the care they provided as a result of the SENSE-GARDEN intervention became more focused on the person with dementia as a unique individual. In reflecting on how care practice is becoming more person-centred, one professional stated:

The more we learn about our residents, the more we can provide care – individually adapted care.

It is not only the time spent together with the person with dementia that helps shift focus onto the individual, but it is also have the opportunity to learn more about the individual. The caregivers felt that building this relationship, it was easier to understand the resident:

So the relation[ship] between the formal carers and the users, it’s much stronger and they could understand them better actually

A similar observation was made with the families of residents with dementia, who reportedly were able to see their relative in a more familiar way:

…the families told us “Oh I didn’t see them in a long time like that” and the interaction between the resident and family member was higher I think in the SENSE-GARDEN than elsewhere.

### Promoting the engagement and well-being of the person with dementia

The professionals felt that SENSE-GARDEN was able to engage people with dementia in a way that benefitted their wellbeing. A caregiver noticed a difference in the behaviour of residents during SENSE-GARDEN sessions compared to being in the care home. She described them in being more wakeful, and thus more positive:

They are not sleeping like they are not in the day centre, they are following a lot, and they have a good feeling and I think that feeling will follow them for the rest of the day

However, the professionals noted that benefits were mainly on short-term memory rather than long-term memory. Across all test sites, the professionals noticed that the people with dementia showed signs of having memory of SENSE-GARDEN, even if they could not explicitly remember their time in the sessions. For example, some residents showed signs of enthusiasm in going towards the SENSE-GARDEN space and somehow knew the route to get to the room. In other instances, residents had positive emotions associated with the SENSE-GARDEN without knowing why:

Even though they didn’t remember they went to SENSE-GARDEN, they wanted to go there, even if they didn’t understand why. So it’s kind of a sign that something is going on there that makes them like to go even if they don’t know where they’re going and it’s strong. I feel that it’s strong.

Residents with dementia at the care homes were attending the SENSE-GARDEN 2–3 times per week, which could perhaps be why they were able to remember the positive feelings associated with being inside the space. This again points to the importance of having a dedicated space inside a care facility where people can engage in leisurely and meaningful activities. Not having the opportunity to visit the SENSE-GARDEN on a regular basis seemed to reverse the positive benefits observed during the intervention period. The professionals at the test sites noticed a decline in the well-being of participants with dementia after the intervention period had ended. They felt that SENSE-GARDEN needs to be continued on a long-term basis if the benefits are going to be sustained:

All our patients…had an improvement on initiation of actions or conversations, but for some of them it didn’t last so long. And we know the improvement decreases after a while and they return to their bad [state] they were with before the SENSE-GARDEN sessions….so we need these sessions for them.

In reflecting on the importance of providing something like SENSE-GARDEN, one professional spoke about how SENSE-GARDEN could be used as a tool to ease the transition to care homes:

“They long for their home. And when they long for their home, their spirit is not that good anymore… I don’t know how to express it, but people change when they get locked up”.

### Giving meaning and value to work

In addition to providing benefits to people with dementia, the SENSE-GARDEN also had a positive impact on the caregivers. The care professionals across the test sites expressed a sense of achievement in being able to provide meaningful activities to residents/patients:

It’s very nice to see when you get a reaction… I have one person that has no facial expression at all… he’s very, very quiet and when I play the music and sometimes he reacts to the music it’s a wow effect for him… once I saw him laughing and it was the best feeling ever for me because now I know yes, he’s following, it’s reaching into him, and he’s having a good time.

I felt useful for the wellbeing and quality of life of the participants (with dementia) and for me it was the most enjoyable… this was the thing that was more important to me

The professionals also enjoyed being inside the SENSE-GARDEN room together with residents. There was some conflict between not having time to facilitate the sessions, yet finding the time investment worthwhile during the sessions:

During those periods of the SENSE-GARDEN sessions, the thought of entering there and staying there it was sometimes…. I don’t know how to say it… something I have to do but I don’t have time for this. But… when you enter the SENSE-GARDEN room you forget about everything and you enjoy with the patient, his own experience that becomes your experience as well.

### Theme 3: Continuous discoveries

The theme “continuous discoveries” represents the ongoing process of learning about the residents/patients, learning what works best for each individual, and professionals’ reflections on challenges and barriers to using SENSE-GARDEN in the long-term. This theme has three subthemes: (1) “learning as an ongoing process” reflecting how the professionals continuously learnt more about each individual as the SENSE-GARDEN sessions progressed, (2) “facilitation” reporting on the professionals’ perspectives on what is important in the facilitation of SENSE-GARDEN sessions, and (3) “addressing barriers to use” reporting challenges such as time consumption, technological issues, and miscommunication within the care home. However, despite these challenges, the professionals felt that the ongoing process behind SENSE-GARDEN enabled continuous discoveries on how they could learn more about residents and patients with dementia. Through investing time into the intervention, the professionals were able to gain insights about what works best for certain individuals and they were able to adapt the sessions – and even the technology itself – to find a solution that best worked for them.

### Learning as an ongoing process

A prerequisite for using SENSE-GARDEN is to first create the ALMA through collecting information from family members. This process in itself can be time consuming, but the professionals felt it was an important part of the process:

If you want to have a very personal SENSE-GARDEN, it takes a lot of time to get good information, pictures… but it is good to have this information

However, the professionals stressed that the learning does not stop after creating the ALMA. The sessions were often adapted as a result of getting to know the person better:

One of the residents, she told me that she was tired of the music I had played and so then (I asked) “Ok, what do you want to hear? And then together we found out that she was very fond of Leonard Cohen and The Beatles, so sometimes it’s not so easy. They don’t remember what they like so we have to adapt after a while and get to know them better

You change [the sessions] all the time because you know that they are fond of something but then you get to know them better and go deeper and find out that they like for example, art and poems, and they don’t always express themselves well in the ALMA report. Maybe there is something coming later and you get to know them better

### Facilitation

The care professionals expressed that the facilitation style of care staff was an important factor in whether the SENSE-GARDEN benefitted the person with dementia. They also noted it was important to be consistent in who is facilitating the sessions, with the suggestion that the same professional should facilitate each session for each individual resident. The skills and enthusiasm of the facilitator were seen as being vital for the session:

I think that the nursing [staff] would like to do that (use SENSE-GARDEN) a lot, but you have to open yourself to using it and not every person can do that. So you really have to know the person who’s going to use it is willing to use it… I think that the quality of a SENSE-GARDEN session is also very dependent on…the one who is leading the session

Additionally, with the intervention being so personal and involving reminiscence, the evocation of negative memories is to be expected. The professionals discussed this in the interviews and emphasised the importance of having a facilitator who knew how to support the person with dementia in processing complex emotions, and who knew to stop the session if needed:

It’s important to be a psychologist or a person who can support in these types of situations when this happens with the participants (with dementia), when they talk about memories that are not positive

Professionals also noted the impact of having family members present during the sessions. They noted the potential for tension or disappointment resulting from the family’s high expectations for the intervention:

There have been times when they saw a picture of something but they didn’t remember anymore and sometimes for the family it was so sad…they wanted the resident to remember but he couldn’t or she couldn’t

### Addressing barriers to use

All test sites addressed issues that they had experienced throughout the course of the intervention. One of the major problems was that of technology. Professionals from the three care homes were particularly disappointed by the SENSE-GARDEN software. The original intention for SENSE-GARDEN was that media contents would be automatically adapted to the preferences of the user, based on feedback given in earlier sessions. The creation of workflows would be automatic, requiring less time for preparation from the care staff. However, this was not the case:

It’s a pity that (the technological issues) have not been resolved. Because in the way that we have SENSE-GARDEN now, we cannot run a session like we wanted to

The ideas are super, but the technology and the way it has been working could be easier

The seamless solution we talked about in the beginning (of the project), it’s not there yet…. It’s a little bit of a disappointment because I thought that everything would get in place by time

Despite these issues, the care professionals still expressed their enthusiasm for the overall concept of SENSE-GARDEN. In some cases, staff facilitating the sessions adopted their own approach to preparing sessions by using software such as Windows Movie Maker or Microsoft PowerPoint to aid the ease of use. However, this raised the concern of over what SENSE-GARDEN actually is:

We can still work with PowerPoint but it’s not actually [pauses]… PowerPoint already works. The thing we’re doing now we still call it SENSE-GARDEN, but we are not really doing SENSE-GARDEN…we have the SENSE-GARDEN room. But we only have the room. We don’t have any of the technological components

Another issue was a lack of communication amongst staff in the care home. In one situation, a resident had visited the SENSE-GARDEN, during which time she and the facilitating member of staff had been talking about the resident’s mother. However, this caused confusion with other members of staff in the care home and resulted in a negative experience for the resident:

She talked a lot about what she had seen when she came back to the nursing home [after the SENSE-GARDEN session] and the nurses there, they hadn’t been in SENSE-GARDEN so they didn’t know what she was talking about… she was asking about her mother and they said “no, she is dead” and she got quite furious… she didn’t sleep well after being SENSE-GARDEN

As a result of this incident, the other members of staff in the care home reportedly said “we don’t think this is good for her” and suggested the resident stop going to SENSE-GARDEN. However, the resident continued attending sessions which “she liked a lot.”

Finally, the professionals acknowledged the fact that SENSE-GARDEN is time consuming and therefore may cause issues with implementation in the future. However, in expressing the importance of maintaining SENSE-GARDEN in the long-term, they suggested potential solutions to make the sessions less time consuming. A common suggestion across test sites was the idea of having more generalized contents in the SENSE-GARDEN in addition to still offering personalized sessions:

You can give some generic pictures and music to people with dementia which almost every person with dementia likes. So I think you can have a generic SENSE-GARDEN and also an individual, unique SENSE-GARDEN and I think in both ways it can be used in care facilities then

We also talked a lot about making sessions that anyone can use like you can choose the 80s or you can choose summer… sessions that are usable for everyone and some sessions that are just for the person

However, with the professionals placing so much emphasis on the importance of knowing the individual and personalizing sessions to each person with dementia throughout the interviews, it is questionable as to whether or not generalized sessions would provide the same benefits found throughout this study e.g. improved understanding and improved relationships. With this being said, there is also the question of whether these benefits were more a result of the technological approach in the SENSE-GARDEN intervention, or more a result of the SENSE-GARDEN being a catalyst for facilitating quality one-to-one time between the professional and the person with dementia. Exploring this further could be a worthwhile topic for future research.

## Discussion

Overall, the experiences from care professionals in this study indicate a potential of SENSE-GARDEN as a beneficial intervention for promoting the well-being of people living with dementia in care, as well as providing staff with an inspiring and meaningful work environment. This is in line with findings of similar work in this area, which has found technology to be useful for engaging care staff in the life story of people with dementia [24, 39]. In the initial interview in 2019, more emphasis was placed on the new space and technical aspects. The staff members were hopeful for the project and, as the project continued, their experiences were positive overall, as reflected in the interviews conducted in 2021. One major limitation reported by all professionals was the issues surrounding technology, thus the SENSE-GARDEN intervention was not as seamless as anticipated. Despite this, most care professionals reflected more on the experience of getting to know the people with dementia. Whilst SENSE-GARDEN was still recognised as a novel and valuable space, it appears that the most important thing to come from the project were the meaningful experiences encountered between staff, residents, and family members. As a result of these meaningful experiences, the professionals in this study felt that their work had more value.

### A potential solution for enhancing job satisfaction and quality of care

With the increasing aging population and an increased need of more health professionals – particularly nurses – it is essential to identify ways of keeping nurses in environments such as care homes. With issues such as staff turnover and lack of staff resources being seen as barriers to implementing person-centred care [21, 22], it is vital that care facilities are developed into inspiring work environments where staff feel that their work has purpose and meaning. As seen in this study, the professionals reported a sense of purpose and achievement from using the SENSE-GARDEN together with residents and patients. Furthermore, a recent review of the needs of nursing staff in providing palliative care for people with dementia include the need for building close relationships, knowing and understanding the person with dementia, and organizational support [40]. Similarly, the recent quality reform for older persons from the Norwegian Ministry of Health and Care Services emphasised the importance of health and care services obtaining information and knowledge of individual backgrounds, interests and life history and using this information to meet the needs of each person, including creating enjoyable moments and meaningful activity in the daily lives of older persons [41]. This study has demonstrated that the SENSE-GARDEN room with the available technology can assist staff in getting to know residents better and building relationships.

Furthermore, whilst the SENSE-GARDEN is mainly discussed in terms of care homes, it is also important to address its potential in hospital environments. Even though one of the test sites in this study was a hospital, the care professionals from this site reported similar findings and reflections as professionals from the care homes. A recent literature review suggested that whilst person-centred care has the potential to improve experiences of hospital staff caring for people with dementia, staff members are often unable to provide such care due to physical needs being prioritised over psychological well-being of patients [42]. This study suggests that a person-centred approach has the potential to improve the experiences of care professionals within a hospital environment. However, further work is needed to assess how feasible it is to sustain this kind of intervention in the long run.

### Addressing barriers to staff-led interventions in practice

Whilst the SENSE-GARDEN provided the professionals in this study an opportunity to deliver more personalized care, all care professionals felt that the technology needs improvement – especially if it is to be used on a long-term basis. The seamless solution of having sessions automatically prepared based on the preferences and interests of the person with dementia was never implemented, and therefore the staff considered the preparation of the sessions to be time consuming.

Common barriers to implementing staff-led interventions in practice are the complexity and the intervention and the staffs’ perceived value of the intervention [21, 43]. This study has shown that care professionals across four different countries all consider the SENSE-GARDEN to be a valuable addition to care practice within their own facilities. Commitment-building has recently been identified as an essential factor for implementing nurse-led interventions in long-term dementia care and the importance of allowing staff to inform and adapt the intervention to situational needs has been emphasised [43]. In this study, the care professionals offered ideas towards how the SENSE-GARDEN could be adapted for future use. Giving staff the option of using SENSE-GARDEN in a flexible and adaptive manner – tailored to the available resources in their care facilities – is essential for implementing its use on a long-term basis outside the context of a research study.

### Implications for future research and practice

Future research on SENSE-GARDEN should first work on making improvements to the technology behind the intervention. The care professionals in this study were disappointed that the technology had not worked as hoped and therefore the preparation of SENSE-GARDEN sessions took up a lot of time. Once the technological aspect is improved, the SENSE-GARDEN should be evaluated further with regards to time consumption and ease of use.

Further work should also explore the use of SENSE-GARDEN in other types of environments. For example, having something similar to SENSE-GARDEN in a person’s private home could provide multiple benefits. People with dementia living at home are normally in milder stages of the disease, meaning that they may be able to take a more active role in preparing the sessions. Family members may also be more involved, especially if living together with the individual with dementia. Building up the contents for sessions could then also become easier. Furthermore, having a prepared user profile with a multitude of media contents and rich information on the person’s life could serve as a useful preparation tool for transitioning into a residential care home. The “SENSE-GARDEN Home” project (AAL-2020-7-720-SCP_SGH) is currently developing a version of the SENSE-GARDEN technology intended for home use. The project is running until November 2021.

In terms of future practice, beyond the context of SENSE-GARDEN, care environments such as care homes and hospitals should strive to adopt person-centred approaches as a means of enhancing the quality of care and improving job satisfaction for professional staff.

## Limitations

Whilst this study benefitted from including interviews with care professionals across 4 different countries, linguistic barriers may have limited the quality of the interviews. The interviews were conducted in English, including one interview where a person immediately translated responses from the native language to English. Thus, data may have been more nuanced had each interview been conducted in the language of the test site. Despite this, we believe that the interviews still captured the main reflections and experiences of the participants.

Another potential limitation to consider is that this study heavily relied on the interpretation and reflections of the first author. However, as Braun and Clarke [44] stress in a recent article on thematic analysis, researcher subjectivity is a resource for knowledge production. Rather than being seen as a “threat to credibility”, the role of researchers in the production of meaning and knowledge should be acknowledged as an interpretive, reflexive process. All data was collected by the first author, who was working for the SENSE-GARDEN project at the time of the study. The advantage of having this author interview all the professionals was that she had a thorough understanding of the SENSE-GARDEN system and had experience of being at all four test sites throughout the project. Therefore, she was able to relate to the care professionals and ensure the interviews were conducted in a conversational style. Moreover, in acknowledging that the researcher is instrumental in the generation of data during semi-structured interviews [45], the interviewer having first-hand experience of using SENSE-GARDEN meant she was able to engage with the respondents in a more affirming and empathetic way, which can generate more detailed responses from interview participants [45]. However, because of the interviewer's role in the project, some respondents may have felt the need to report positively on the intervention. The interviewer assured the respondents that they could express negative opinions in the interviews and most participants did voice their concerns, especially regarding the technology. The first author also led the analysis of transcripts, and her interpretation of the data may have been influenced by her role in the project. However, two of the four authors of this paper are not affiliated with the SENSE-GARDEN project and therefore were able to remain more neutral throughout the analysis and reporting of the data. Despite the high level of involvement of researchers affiliated with the SENSE-GARDEN project, our ultimate goal is to contribute to supporting staff in creating meaningful activities in care. Therefore, we aimed to present SENSE-GARDEN in a way that accurately reflected the care professionals’ beliefs and experiences of the intervention.

Finally, it should be acknowledged that all care professionals in this study were women. This imbalance in gender is common amongst dementia studies, in which most carers are women.

## Conclusions

 This paper has presented the experiences of care professionals across Norway, Portugal, Belgium and Romania in their use of SENSE-GARDEN. The key messages from the study are presented in Table 2.

**Table 2:** Key messages

KEY MESSAGES
• **Barriers** Barriers to using technology for supporting meaningful activities in care include the high cost of equipment, the time-consumption of preparing sessions, and the length of time it takes to get to know people with dementia – especially those in later stages of the disease. Issues with the technology itself can also create another barrier to use.
• **Lessons learned** It is possible to deliver meaningful activities in dementia using technology, but barriers first need to be overcome if this kind of technology is going to be implemented on a long-term basis. Similar studies should ensure that the technology works as intended and is usable prior to being tested in practice.
• **Recommendations for future research and practice** People in later stages of dementia are not “closed books”; they are individuals with rich life stories. These stories should be used as resources for care professionals to better know the person with dementia, helping to improve relationships and job satisfaction within dementia care practice. Future research should identify feasible ways of implementing activities that utilize these stories on a long-term basis.

Overall, SENSE-GARDEN is a promising intervention in helping alter staff perceptions of individuals with dementia, improving communication between staff and residents/patients, and creating opportunities for meaningful activities and the delivery of person-centred care. The SENSE-GARDEN is an innovative way of conveying and learning about the unique life stories of people with dementia. It also enables care staff to interact with and build upon these stories when engaging in activities together with the resident inside the SENSE-GARDEN. Whilst the preparation and facilitation of sessions is time consuming, the care professionals in this study consider that the benefits and sense of achievement that comes from using SENSE-GARDEN makes this time investment worthwhile. However, if the intervention is going to be used on a long-term basis, the technological aspects of the intervention must first be improved and less time-consuming approaches to using the SENSE-GARDEN must be explored

## Supplementary information

**Additional file 1.**

## Data Availability

The datasets generated and analysed during the current study are available in the Zenodo repository, 10.5281/zenodo.4679428.

## Abbreviations

ALMA: Arts of Life Memory Album
RTA: Reflexive Thematic Analysis
