# Supplementary material for: Towards personalized dementia care through meaningful activities supported by technology: A multisite qualitative study with care professionals
Source: BMC Geriatr. 2021 Aug 21;21:468. doi: 10.1186/s12877-021-02408-2 (PMC8380345; doi:10.1186/s12877-021-02408-2)
Supplement: Supplementary file 1 — Additional file 1. [file 12877_2021_2408_MOESM1_ESM.docx]

**Supplementary material for “Towards personalized dementia care through meaningful activities supported by technology: A multisite qualitative study with care professionals”**

**Gemma Goodall^1^, Kristin Taraldsen^2^, Randi Granbo^2^, J Artur Serrano^2^**

^1^Department of Mental Health, Norwegian University of Science and Technology, Trondheim, Norway

^2^Department of Neuromedicine and Movement Science, Norwegian University of Science and Technology, Trondheim, Norway

**Corresponding author:** Gemma Goodall, [gemma.goodall@ntnu.no](mailto:gemma.goodall@ntnu.no)

**Supplementary material A: Interview guide for Part 1 of the study**

1. What were your initial reactions to seeing the SENSE-GARDEN for the first time?
2. Do the residents talk about the new SENSE-GARDEN space?

- What were their initial reactions to seeing the physical structure of the SENSE-GARDEN in the care home?

1. Can you share an example of a positive experience you have had in the SENSE-GARDEN?
2. Have you had any negative experiences in the SENSE-GARDEN?
3. Have you learnt anything new about the residents as a result of using SENSE-GARDEN?

- What do you think about the potential of SENSE-GARDEN being used to help staff to get to know residents better?
- Do you think it can help new members of staff to get acquainted with the residents?

1. How does using the SENSE-GARDEN with a resident make you feel?
2. What do you think makes a SENSE-GARDEN visit “successful”? (Prompts below if needed)

- Is it the conversation?
- Is it the ability to recall memories?

1. How does using the SENSE-GARDEN with a resident fit into your daily working routine?

- Do you see more benefits or difficulties to your work? Can you give examples?

1. How is using SENSE-GARDEN different to other leisure activities in the care home, such as, for example, group music sessions in the activity room?
2. How do you think the experience, or the space, could be made better?
3. Do you have any other comments?

**Supplementary material B: Interview guide for Part 2 of the study**

1. Could you describe your overall experience of using SENSE-GARDEN?

2. Can you share an example of a positive experience you have had in the SENSE-GARDEN?

3. Have you had any negative experiences in the SENSE-GARDEN?

4. How did the residents react during the SENSE-GARDEN sessions?

5. Have you noticed any change in the residents’ behaviour outside of the sessions?

1. Do the residents talk about the SENSE-GARDEN space outside of the sessions?
2. What is your approach to planning the SENSE-GARDEN sessions?

a. In what way were the family members involved

b. Prompt: ALMA questionnaire

c. Has the resident asked for specific media contents?

1. Have you been in a situation where a resident becomes upset by the media contents?

a. How do you handle the situation?

1. Have you learnt anything new about the residents as a result of using SENSE-GARDEN?

a. What do you think about the potential of SENSE-GARDEN being used to help staff to get to know residents better?

b. Do you think it can help new members of staff to get acquainted with the residents?

1. What results or outcomes would you like to see (or consider most important) during/after a session? (Prompts below if needed)

a. Improved conversation?

b. The ability to recall memories?

c. Other aspects?

1. How does using the SENSE-GARDEN with a resident make *you* feel?
2. How does using the SENSE-GARDEN with a resident fit into your daily working routine?

a. Do you see more benefits or difficulties to your work? Can you give examples?

13. How is using SENSE-GARDEN different or connected to other leisure activities in the care home?

14. Do you think SENSE-GARDEN could be used in care on a long-term basis?

15. How do you think the experience or the space could be made better?

16. Do you have any other comments?
